# Estrogen metabolites in premenopausal women living in China, Mongolia, and the United Kingdom

**DOI:** 10.1186/s12885-026-16169-x

**Published:** 2026-06-10

**Authors:** Lauren C. Houghton, Davaasambuu Ganmaa, Isabel Dos Santos Silva, Roni Falk, Michelle L. Lui, Kathleene T Ulanday, Gretchen Gierach, Xia Xu, Dambadarjaa Davaalkham, Wei Zheng, Rebecca Troisi

**Affiliations:** 1 Columbia University Mailman School of Public Health, New York, USA; 2 Departments of Nutrition and Epidemiology, Harvard School of Public Health, Boston, MA USA; 3 Health Sciences University of Mongolia, Ulaanbaatar, Mongolia; 4 Department of Non-communicable Disease Epidemiology, London School of Hygiene and Tropical Medicine, London, England; 5 Division of Cancer Epidemiology and Genetics, National Cancer Institute, National Institutes of Health, Rockville, MD USA; 6 Frederick National Laboratory for Cancer Research, Cancer Research Technology Program, Leidos Biomedical Research, Inc., Frederick, MD USA; 7 Vanderbilt-Ingram Cancer Center, Vanderbilt Epidemiology Center, Vanderbilt, Nashville TN, USA

**Keywords:** Estrogen metabolism, Breast cancer, International incidence rates, Asia, Mongolia, China, U.K

## Abstract

**Background:**

Breast cancer incidence is lower in women living in Asia than women living in North America and Northern Europe. However, large differences exist within Asia, for example, Mongolia’s breast cancer rate is even lower than China’s. Endogenous estrogens in Asian populations have been hypothesized to explain the disparity because of their strong positive correlations with breast cancer, though few studies have compared estrogen metabolites (EM) within Asia or between Asia and higher breast cancer risk countries.

**Methods:**

We measured urinary concentrations of estradiol and estrone (parent estrogens) and 13 EM (non-parent estrogens) formed by irreversible hydroxylation at the C-2, C-4, or C-16 positions of the steroid ring in premenopausal women living in Ulaanbaatar, Mongolia (*n* = 282), Shanghai, China (*n* = 121), and the United Kingdom (*n* = 125). We performed analysis of variance (ANOVA) using a generalized linear model procedure to assess differences in hormone concentrations between British and Asian (Chinese and Mongolian), and Chinese and Mongolian. Exponentiated least square means were used to calculate percent differences. We compared differences in overall means adjusted for age, body mass index, and parity by country during the follicular, peri-ovulatory, and luteal phases.

**Results:**

Total, parent, and non-parent estrogen concentrations were generally higher in British than Asian women, while the non-parent to parent ratio was lower. While parent estrogens appeared higher in Chinese compared with Mongolian women, differences did not reach statistical significance except for peri-ovulatory estrone; total estrogen metabolites in the 2-, 4-, and 16-pathways were higher in Mongolian than Chinese women. The non-parent to parent ratio was highest in Mongolian women followed by Chinese women, with British women having the lowest ratio.

**Conclusion:**

Total urinary estrogens, both parent and their metabolites, were higher in British compared with Asian women. Our data showing the ratio of non-parent to parent estrogens, possibility reflecting greater estrogen metabolism, in women living in Mongolia, with one of the lowest breast cancer rates globally, than in women living in China, and in Chinese compared with British women, are consistent with their relative breast cancer risks and suggestive of a potentially protective metabolic profile.

**Supplementary Information:**

The online version contains supplementary material available at 10.1186/s12885-026-16169-x.

## Background

Postmenopausal, and to some extent premenopausal, breast cancer incidence rates in Asian countries are substantially lower than in the United States (U.S.) and European countries [1–3]. These differences may be explained by hormonal mechanisms; specifically, lower estrogen concentrations in Asian populations than US populations are consistent with a generally positive association between estrogens and breast cancer risk. While descriptive studies have demonstrated lower circulating estradiol levels in Chinese than U.S. women, the magnitude of the difference appears insufficient to fully explain the differences in their breast cancer risk [4]. Moreover, our observation that circulating estradiol is *not* lower in premenopausal Mongolian women (who have a lower breast cancer incidence rate than even the Chinese) compared with British women indicates inconsistency in the generalization that Asians have reduced estrogen concentrations [5].

The association of endogenous estrogens with breast cancer risk may depend on when estrogens are measured (pre- vs. postmenopause, and menstrual cycle phase) and menopausal status at cancer diagnosis. Some studies show that the association with premenopausal breast cancer risk is limited to estradiol measured in the follicular phase [6]. In addition, studies of estrogen metabolism in urine and breast cancer risk have been less consistent [7, 8] than studies measuring circulating estradiol in serum or plasma [6, 9].

There is evidence that differences in estrogen metabolism—the irreversible hydroxylation of estrone and estradiol into 13 metabolites—is associated with breast cancer risk. Studies in U.S. populations indicate that breast cancer risk may depend on the extent to which hydroxylation occurs along the 2, 4, or 16 pathway [10]. Higher concentrations of estrogen metabolites (EM) and more extensive 2-hydroxylation are associated with lower risk among both U.S. and Chinese postmenopausal women [10]. The role of estrogen metabolism in premenopausal breast cancer risk is less clear, partly due to wide variation in concentrations through the menstrual cycle. Migrant data have shown that premenopausal women who migrated to the U.S. from China, Japan, and the Philippines had lower urinary 2- and 16- metabolites and their ratio, than Asian women born in the U.S [11].

Within Asia, breast cancer rates are lower in limited resource countries than higher resource countries, and in rural compared with urban areas [12, 13]. For example, incidence in Mongolia is one of the lowest in the world (6.6/100,000) while China, its neighbor to the south, has about three times this rate (18.7/100,000). Studying Mongolian as well as Chinese populations can address ecologically the consistency of endogenous hormone levels among several populations at various breast cancer risks.

We compared concentrations of urinary estradiol, estrone, and 13 estrogen metabolites among premenopausal British, Chinese, and Mongolian women to ascertain whether differences are consistent with a role of estrogen metabolism in explaining breast cancer rates in the respective countries. The study did not include individual-level information on cancer incidence. We hypothesize that estrogen metabolism would not only differ between U.K. and Asian (Chinese and Mongolian) women, but also between women living in higher and lower resource Asian countries (China and Mongolia).

## Methods

The ethical review boards at Mongolia’s Ministry of Health, the National University of Mongolia, the United States (U.S.) National Cancer Institute (National Institutes of Health), the Harvard School of Public Health, Vanderbilt University, and the London School of Hygiene and Tropical Medicine approved this study. All participants provided written, informed consent, and study procedures were conducted in accordance with the ethical standards of the institutional and national research committees and with the 1964 Declaration of Helsinki and its later amendments.

### Data collection

#### Mongolia

In the spring of 2009, study staff recruited mothers of school children, who were 45 years of age or younger, and who were not pregnant or breastfeeding, from two primary schools in Ulaanbaatar, Mongolia. Of the 420 invited women, 100% agreed to participate: 199 from a school in the city’s outskirts, and 221 from a school in the city center. We excluded women who were currently using oral contraceptives leaving 282 participants in the analytical dataset.

Study staff interviewed participants about demographics, migration, and medical history using questionnaires designed for the original aims of the study (See Supplementary Materials) and measured weight and height. Participants donated spot urine samples that study staff put on ice until they could be stored without preservatives at − 70 °C. Participants reported date of first day of last menstrual period and later confirmed the first day of the next menstrual period.

#### China

We selected study participants from the Shanghai Women’s Health Study, a population-based cohort of 75,221 women aged 40 to 70 years and enrolled between 1997 and 2000 for which methods have been previously described [14]. Of those women who completed the survey, 279 were younger than age 40 at the time of the baseline interview allowing for us to include younger women in the current analytical sample. Among premenopausal women not using hormone replacement therapy at the time of sample collection, study staff randomly selected a sample of 121. Trained interviewers assessed participant characteristics using the study questionnaires publicly available here https://swhs-smhs.app.vumc.org. Most (88%) participants donated a spot urine into a cup containing 125 mg of ascorbic acid. Study staff measured participants’ weight and height. The cup was kept cold, processed within six hours of collection, and stored at -70 °C. Participants were followed using biennial home visits. Participants provided the number of days between their last menstrual period and sample collection.

#### Britain

A sample of women from Britain who identified as white were recruited nationally between November 2008 and September 2009 to serve as controls for the British Breast Cancer Study, a case-control study on the genetics of breast cancer [15, 16]. These women were unaffected friends nominated by each case. For the present study, we chose 150 controls randomly; of these, we included 125 women who were ≤ 50 years of age and not pregnant, breastfeeding or currently using oral contraceptives. Study staff collected self-reported height and weight via questionnaire designed to answer the original aims (see Supplementary Material). For hormonal assays, participants donated urine samples targeting specific days of their cycle. Sampling days were the 3 days immediately preceding the predicted day of ovulation, the predicted ovulation day, and the first, second, and seventh days after predicted ovulation. We selected one sample that was stored without preservative from each woman for this study. Women provided the first day of their most recent menstrual period and usual cycle length and returned a form in a pre-paid envelope indicating the first day of their next period.

### Hormone assays

The Laboratory of Proteomics and Analytical Technologies, Cancer Research Technology Program, Leidos Biomedical Research, Inc. (Frederick, MD, USA) quantified the estrogen metabolome in the urine samples using previously described methods [17]. The laboratory was blinded to country of origin of the sample. They used stable isotope dilution LC-MS/MS to measure estrone, estradiol, and 13 estrogen metabolites in 500 ml of urine. In urine, parent estrogens and their metabolites are present primarily in conjugated form. They used a Helix pomatia preparation to remove glucuronide and sulfate moieties, allowing measurement of total amounts for each estrogen/estrogen metabolite. To estimate absolute concentrations, they used six stable, isotopically labeled internal standards: 13 C-labelled estrone and estradiol and 2 H-labelled 2-hydroxyestradiol, 2-methoxyestradiol, estriol, and 16-epiestriol. In each assay batch, they constructed calibration curves by plotting estrogen/estrogen metabolite: isotopically labeled standard peak area ratios vs. known amount of each estrogen/estrogen metabolite. They then interpolated the quantity of estrogen/estrogen metabolite by a linear function. They standardized molar quantities to creatinine [18]. We measured assay reliability using 3 blinded replicates from 7 individuals for a total of 21 quality control samples. Each estrogen/estrogen metabolite had an average coefficient of variation of < 5%.

We analyzed estrogens and estrogen metabolite individually and in groups representing metabolic pathways Estrone and estradiol, the parent estrogens, are irreversibly hydroxylated at the C-2, C-4, or C-16 positions of the steroid ring, leading to a cascade of metabolites. The combined metabolites in each of the C-2, C-4 and C-16 pathways allowed us to investigate whether patterns of estrogen metabolism differed in the three populations (Chinese; Mongolian; British). These included metabolites in the 2-hydroxylation pathway (2-pathway, i.e., 2-hydroxyestrone, 2-methoxyestrone, 2-hydroxyestradiol, 2-methoxyestradiol, and 2-hydroxyestrone-3-methyl ether); metabolites in the 4-hydroxylation pathway (4-pathways, i.e., 4-hydroxyestrone, 4-methoxyestrone, and 4-methoxyestradiol); and metabolites in the 16-hydroxylation pathway (16-pathway, i.e., 16α-hydroxyestrone, estriol, 17-epiestriol, 16-ketoestradiol, and 16-epiestriol). Total metabolites were the sum of 2-pathway, 4-pathway and 16-pathway; and total estrogens were defined as the sum of the parent estrogens and metabolites. We included the 2:16 ratio because the ratio can change even if the differences in the 2-pathway and 16-pathway metabolites are in the same direction. We also examined the ratio of non-parent estrogens to the sum of parent estrogens (estradiol and estrone).

### Statistical analysis

We compared the distribution of demographic and lifestyle characteristics for the three samples (Mongolian, Chinese, and British). We truncated the menstrual cycle at 28 days because of small numbers of women with longer cycles (*n* = 3 in Mongolia; *n* = 10 in U.K.). Least square means of follicular, peri-ovulatory, and luteal phase hormones were estimated from analyses including menstrual period days 0–11, 12–16 and 17–28, respectively. We stratified all models by menstrual phase. We performed an analysis of variance (ANOVA) using a generalized linear model procedure with logarithm-transformed hormone values to assess differences in hormone concentrations between samples, first comparing British and Asian (Chinese and Mongolian), and then Chinese and Mongolian. We used the exponentiated least square means to calculate percent differences for each menstrual phase. We also ran all pairwise comparisons. The basic model (Model 1) adjusted for age and menstrual cycle day, and another model (Model 2) additionally adjusted for body mass index (BMI) and parity. The basic model (Model 1) was repeated restricted to parous women and restricted to non-smoking women.

The large number of estrogen metabolites, the necessity of assessing differences among the study populations by menstrual phase, and the additional subgroup analyses could have resulted in increased type I errors. Therefore, we evaluated the findings more by observing patterns than relying on statistical significance and treated the findings as hypothesis generating.

## Results

### Sample characteristics

The mean age of Chinese women was 40.2 years with 77% of women aged 40 and over, 38.3 years in British women (42%), and 35.2 years in Mongolian women (23%) (Table 1). All Mongolian women were parous (by study design – mothers of school children), while 95% of the Chinese women, and about two-thirds of the British women were parous. Average BMI was similar in British and Mongolian women (BMI 25.62 and 24.94 kg/m^2^, respectively), and lower in Chinese women (BMI 22.27 kg/m^2^). Almost none of the Chinese women reported having ever smoked (3.2%), while 16.7% of Mongolians and 34.4% of British women reported having smoked. The distribution of cycle day differed in British women compared to Asian women; a substantially higher proportion of British women provided their urine specimen during the peri-ovulatory period (67%) compared to both Chinese and Mongolian women (12–13%).

**Table 1:** Descriptive statistics for Mongolian, Chinese, and British women

Characteristic	Mongolian (*n* = 282)		Chinese (*n* = 121)		British (*n* = 125)	
	*n*	%	*n*	%	*n*	%
Menstrual Phase						
Follicular (days 0–11)	132	47%	58	48%	31	25%
Peri-Ovulatory (days 12–16)	37	13%	14	12%	84	67%
Luteal (days 17–28)	113	40%	49	40%	10	8%
Age (at urine collection; years)						
24–29	34	12%	6	5%	4	3%
30–34	102	36%	11	9%	19	15%
35–39	81	29%	11	9%	49	39%
40+	65	23%	93	77%	53	42%
Body mass index (kg/m2)						
Missing	0	0%	0	0%	12	10%
< 20	20	7%	24	20%	6	5%
20 - <25	133	47%	80	66%	57	46%
25 - <30	89	32%	16	13%	33	26%
30+	40	14%	1	1%	17	14%
Parity / number of live births						
Missing	0	0%	0	0%	2	2%
0	0	0%	6	5%	40	32%
1	80	28%	110	91%	17	14%
2	117	41%	5	4%	53	42%
3+	85	30%	0	0%	13	10%
Ever smoked	50	18%	3	2%	43	34%

Adjusted least square means for all the estrogens are presented in Supplemental Table 1. Comparisons are made using percentage differences in means.

### Hormone comparisons between British and Asian women

**Fig. 1:**
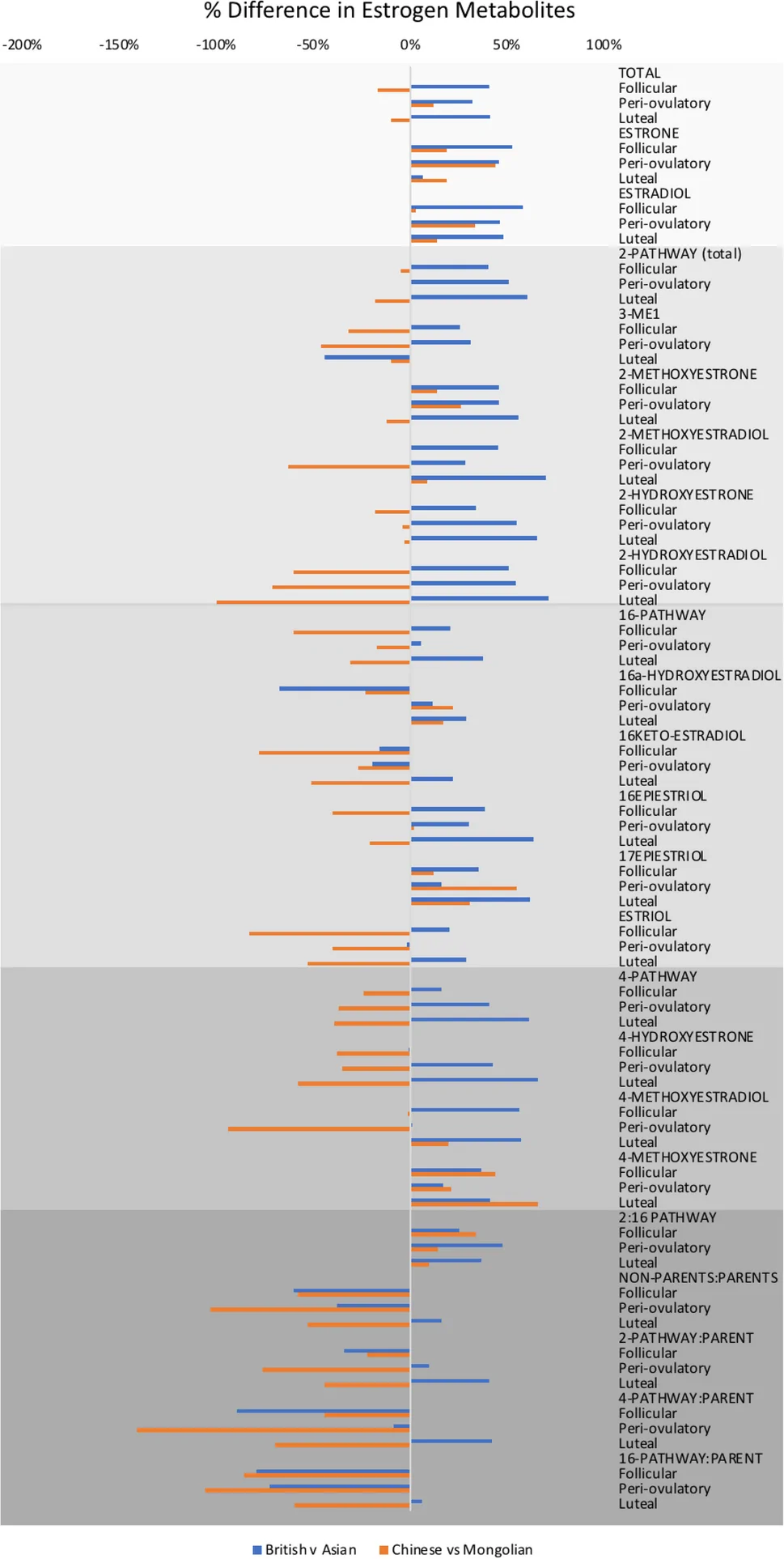
Percent differences in mean estrogens between British and Asian women and Chinese and Mongolian women

In general, mean estrogen and estrogen metabolite concentrations among British women were higher than among Asian women (Table 2; Fig. 1). Adjustment for BMI and parity did not affect hormone differences between British and Asian women in the peri-ovulatory and follicular phases; differences that were observed in the luteal phase were likely due to smaller sample size.

**Table 2:** Mean differences in estrogen metabolites between British and Asian women, and Mongolian and Chinese women

	Model 1 adjusted for age and cycle day				Model 2 adjusted for age, cycle day, BMI and parity			
	British v Asian		Chinese vs. Mongolian		British v Asia		Chinese vs. Mongolian	
	% Difference^a^	*P* value	% Difference^b^	*P* value	% Difference^a^	*P* value	% Difference^b^	*P* value
Total								
Follicular	38%	<0.001	-13%	0.25	41%	<0.001	-17%	0.21
Peri-ovulatory	32%	<0.001	15%	0.44	32%	<0.01	12%	0.56
Luteal	28%	0.13	-7%	0.56	41%	0.04	-10%	0.46
Estrone								
Follicular	50%	< 0.0001	22%	0.06	53%	< 0.0001	19%	0.16
Peri-ovulatory	49%	< 0.0001	46%	0.01	46%	< 0.0001	44%	0.03
Luteal	5%	0.86	22%	0.09	6%	0.83	19%	0.19
Estradiol								
Follicular	56%	< 0.0001	5%	0.71	58%	< 0.0001	3%	0.83
Peri-ovulatory	49%	< 0.0001	35%	0.08	47%	< 0.0001	33%	0.12
Luteal	39%	0.06	16%	0.22	48%	0.03	14%	0.33
2-Pathway (total)								
Follicular	38%	<0.01	-1%	0.92	41%	<0.001	-5%	0.72
Peri-ovulatory	49%	< 0.0001	4%	0.85	51%	< 0.0001	0%	0.99
Luteal	54%	<0.01	-14%	0.33	61%	<0.01	-18%	0.27
3-ME1								
Follicular	27%	0.09	-26%	0.13	26%	0.12	-32%	0.10
Peri-ovulatory	31%	0.03	-38%	0.27	31%	0.03	-46%	0.21
Luteal	-22%	0.51	-5%	0.78	-44%	0.31	-10%	0.58
2-methoxyestrone								
Follicular	44%	<0.001	16%	0.21	46%	<0.001	14%	0.34
Peri-ovulatory	45%	< 0.0001	28%	0.23	46%	0.0001	26%	0.30
Luteal	47%	0.02	-9%	0.56	56%	0.02	-12%	0.51
2-methoxyestradiol								
Follicular	47%	0.02	2%	0.94	46%	0.04	0%	0.99
Peri-ovulatory	28%	0.17	-60%	0.28	28%	0.19	-63%	0.28
Luteal	63%	0.03	10%	0.67	70%	0.03	9%	0.74
2-hydroxyestrone								
Follicular	32%	0.04	-15%	0.34	34%	0.03	-18%	0.31
Peri-ovulatory	52%	< 0.0001	-1%	0.97	55%	< 0.0001	-4%	0.88
Luteal	60%	<0.01	0%	0.98	66%	<0.01	-3%	0.87
2-hydroxyestradiol								
Follicular	44%	<0.01	-59%	<0.01	51%	<0.001	-60%	<0.01
Peri-ovulatory	49%	< 0.0001	-69%	0.08	55%	< 0.0001	-71%	0.08
Luteal	63%	<0.01	-99%	< 0.0001	71%	<0.001	-100%	<0.001
16-pathway								
Follicular	19%	0.20	-59%	<0.001	21%	0.16	-60%	0.001
Peri-ovulatory	2%	0.89	-16%	0.55	6%	0.68	-17%	0.55
Luteal	19%	0.43	-30%	0.05	37%	0.13	-31%	0.08
16a-hydroxyestradiol								
Follicular	22%	0.19	-6%	0.72	-68%	0.30	-23%	0.22
Peri-ovulatory	17%	0.27	35%	0.15	11%	0.48	22%	0.42
Luteal	23%	0.40	29%	0.04	29%	0.35	17%	0.30
16keto-estradiol								
Follicular	-19%	0.30	-63%	<0.001	-16%	0.40	-78%	<0.001
Peri-ovulatory	-29%	0.09	-14%	0.61	-19%	0.25	-27%	0.38
Luteal	16%	0.53	-38%	0.03	22%	0.44	-51%	0.01
16EpiEstriol								
Follicular	37%	<0.01	-39%	0.01	38%	<0.01	-40%	0.02
Peri-ovulatory	30%	0.01	3%	0.91	30%	0.02	2%	0.93
Luteal	44%	0.03	-21%	0.18	64%	<0.001	-21%	0.23
17EpiEstriol								
Follicular	38%	0.04	26%	0.09	35%	0.06	12%	0.51
Peri-ovulatory	20%	0.25	62%	<0.01	16%	0.39	55%	0.03
Luteal	61%	0.01	42%	<0.01	62%	0.03	31%	0.08
Estriol								
Follicular	17%	0.29	-93%	< 0.0001	20%	0.21	-83%	< 0.0001
Peri-ovulatory	-10%	0.55	-49%	0.13	-2%	0.91	-40%	0.22
Luteal	2%	0.96	-62%	<0.001	29%	0.31	-53%	0.01
4-pathway								
Follicular	15%	0.34	-11%	0.44	16%	0.32	-24%	0.16
Peri-ovulatory	40%	<0.001	-21%	0.48	41%	<0.001	-37%	0.26
Luteal	44%	0.03	-25%	0.14	61%	<0.01	-39%	0.05
4-hydroxyestrone								
Follicular	-2%	0.90	-23%	0.18	-1%	0.96	-38%	0.07
Peri-ovulatory	42%	<0.01	-18%	0.58	43%	<0.01	-35%	0.35
Luteal	45%	0.06	-41%	0.04	66%	<0.01	-58%	0.02
4-methoxyestradiol								
Follicular	55%	<0.01	-3%	0.88	57%	<0.01	-1%	0.96
Peri-ovulatory	5%	0.82	-97%	0.09	1%	0.96	-94%	0.11
Luteal	54%	0.06	18%	0.37	57%	0.81	20%	0.38
4-methoxyestrone								
Follicular	40%	0.01	47%	0.28	37%	0.03	44%	0.28
Peri-ovulatory	6%	0.72	11%	0.09	17%	0.30	21%	0.09
Luteal	28%	0.32	56%	0.04	41%	0.13	66%	0.04
Pathways and Ratios								
2:16 Pathway								
Follicular	24%	0.09	36%	<0.001	25%	0.81	34%	<0.01
Peri-ovulatory	48%	< 0.0001	17%	0.44	48%	< 0.0001	14%	0.55
Luteal	44%	0.03	13%	0.34	37%	0.14	10%	0.51
Non-parent: parent								
Follicular	-56%	0.0001	-59%	< 0.0001	-60%	< 0.0001	-58%	< 0.0001
Peri-ovulatory	-51%	< 0.0001	-105%	< 0.0001	-38%	<0.01	-103%	< 0.0001
Luteal	4%	0.81	-55%	< 0.0001	16%	0.42	-53%	< 0.0001
2-pathway: Parent								
Follicular	-33%	0.02	-21%	0.05	-34%	0.02	-22%	0.07
Peri-ovulatory	0%	0.98	-75%	<0.01	10%	0.34	-76%	<0.01
Luteal	38%	0.02	-44%	<0.001	41%	0.03	-44%	<0.01
4-pathway: Parent								
Follicular	-82%	<0.001	-33%	0.03	-90%	<0.001	-44%	0.02
Peri-ovulatory	-17%	0.29	-120%	<0.01	-8%	0.60	-141%	<0.001
Luteal	25%	0.31	-57%	<0.01	42%	0.09	-70%	0.001
16-pathway: Parent								
Follicular	-74%	0.0001	-90%	< 0.0001	-79%	< 0.0001	-86%	< 0.0001
Peri-ovulatory	-91%	< 0.0001	-112%	<0.001	-73%	< 0.0001	-106%	0.001
Luteal	-10%	0.67	-64%	< 0.0001	6%	0.82	-60%	<0.001

^a^% difference = (e^(British) – e^(Asian)) / (e^(British))

^b^% difference = (e^(Chinese) – e^(Mongolian)) / (e^(Chinese))

We describe results from the models adjusted for age, cycle day, BMI, and parity in Table 2. Total estrogens (32%-41% difference), estrone (46%-53%), and estradiol (47%-58%) were higher in British women than Asian women in the follicular and peri-ovulatory phases, as were total metabolites. 2-pathway estrogen concentrations were generally higher in British women than Asian women across all three menstrual phases (41–61%). Follicular and luteal phase 16-pathway metabolite concentrations also were generally higher in British than Asian women (21–37%) as were 4-pathway metabolite concentrations (16–61%). In contrast, the non-parent to parent ratio (38%-60%) was lower in the British women than Asian women in the follicular and peri-ovulatory phases.

### Hormone comparisons between Chinese and Mongolian women

Differences between Mongolian and Chinese women (Table 2) varied by pathway and metabolite. Adjustment for BMI and parity did not affect most of the hormone differences between Mongolian and Chinese women although some of the results for 16-pathway and 4-pathway metabolites were strengthened. Parent estrogens appeared greater in Chinese than Mongolian women, however, only estrone was statistically significantly elevated and only in the peri-ovulatory phase (44%-46%). Except for higher 2-hydroxyestradiol in Mongolian women than Chinese women in all phases (60–100% difference), 2-pathway metabolite concentrations were similar. 16-pathway metabolites were higher in Mongolian than Chinese women (40%-83%) mainly in the follicular phase, except for peri-ovulatory and luteal 17EpiEstriol, which were 31–55% higher in Chinese than Mongolian women. 4-pathway metabolite concentrations, except for 4-methoxyestrone, also were generally higher in Mongolian than Chinese women but only luteal phase 4-hydroxyestrone reached statistical significance. The ratio of non-parent to parent estrogens was lower among Chinese compared to Mongolian women, up to two-fold in the peri-ovulatory phase (103%-105%).

### Differences by menstrual cycle phase

The differences in estrogen concentrations among groups generally were consistent over the menstrual cycle. Any differences tended to be between the peri-ovulatory or luteal phase. Interestingly, estriol was significantly lower during the follicular phase but higher during the luteal in British women compared with Asian women.

Restricting the analysis to parous women yielded similar results (data not shown), except in the 4-pathway. Similarly, restricting the analysis to non-smoking women yielded similar results except for 4-methoxyestrone; mean differences in luteal phase 4-pathway metabolites were greatly reduced and not statistically significant between UK and Mongolian and Chinese women (data not shown).

## Discussion

Our study including women from historically low and high breast cancer risk populations demonstrated a pattern of greater total and parent estrogens and estrogen metabolite concentrations in women living in the U.K. versus women living in Asia. Women living in China appeared to have slightly higher estrogens, overall, than those living in Mongolia, but differences did not reach statistical significance. Mongolian women, however, had greater concentrations of estrogen metabolites, mainly in the 16-pathway than Chinese women. Higher concentrations of total and parent estrogens in British than Asian women is consistent with the positive association of circulating estrogens with breast cancer risk. The ratio of non-parent to parent estrogens, reflecting the extent of estrogen metabolism, was highest among the Mongolian women, followed by the Chinese women and the British women. Although we did not evaluate breast cancer and can only comment on associations, this pattern is consistent with results of studies among U.S. and Chinese postmenopausal women showing greater estrogen metabolism associated with reduced breast cancer risk [10], and with lower breast cancer rates in Mongolia than China, and in China than the U.K.

Although the differences we observed in hormones in the current study would be unlikely to fully explain the large difference in breast cancer incidence rates between low- and high-risk populations, they do raise questions regarding previous associations of estrogen metabolism and breast cancer found in U.S. nested case-control studies. For example, the Nurses’ Health Study observed an inverse association between urinary estrogens and breast cancer risk [7, 8] which is perplexing given that circulating estrogens are positively associated with risk [9, 19]. Serum and urinary estrogens are not highly correlated [20] indicating that urinary estrogens measure different biological activity than estrogens measured in serum and that higher levels of excreted estrogens do not simply reflect lower amounts in circulation. While we standardized estrogens to creatinine level to account for differences in kidney function, metabolite ratios reflect not only metabolic conversion but also renal clearance, hydration status, and other physiological factors. Our study suggests that additional studies of estrogen metabolism and breast cancer risk are needed in diverse populations using values in serum.

The International Agency for Research on Cancer (IARC) bases Mongolia’s breast cancer incidence figures on national registry information, which carries a D5 quality rating. Although limitations in case reporting may partially contribute to the low incidence observed, such data issues are unlikely to account for the substantial difference in incidence between Mongolia and China. In addition, Mongolia is experiencing dramatic socio-economic changes; as a result, there may be strong cohort effects in breast cancer incidence – i.e., current rates mainly reflect incidence at older ages. The women in the present study were premenopausal and their future breast cancer incidence rates are likely to be higher than the current rates possibly affecting the generalizability of our results. However, this phenomenon, increases in resources that affect lifestyle factors that may be associated with cancer, is occurring in other countries in transition as well, such as China. Future differences in breast cancer incidence may be smaller between countries in transition but are likely to remain.

A major strength of our study was our ability to compare hormones across the entire menstrual cycle. Because estrogen concentrations vary substantially over the cycle, sampling on the same cycle day (often day 21 to capture the mid-luteal peak) for all participants is ideal for minimizing variation between samples but is logistically challenging. Instead, we sampled women without regard to cycle day and compared estrogen concentrations across follicular, peri-ovulatory and luteal phases adjusting for cycle day. 

There were limitations to our study. Our interpretation of hormone differences between countries assumes that the populations sampled are representative of the women living in China, Mongolia, and Britain. The sample of British women were born in areas throughout the country. This was also true for the Mongolian sample; the women born outside of Ulaanbaatar were from all but two of the country’s provinces. The Chinese women, however, may be less representative as they were from Shanghai only, a large urban city. While we measured estrogen metabolites in premenopausal women, the largest international differences in breast cancer rates relate to the postmenopausal period. However, there are substantial differences in rates between premenopausal women as well [2], and less data on hormones because of the challenges of considering the menstrual cycle. Furthermore, much of the speculation about the cumulative estrogen dose effects on breast cancer risk pertains to the period before menopause, as cumulative estrogen exposure is largely driven by the premenopausal period because of its higher baseline concentrations.

The large number of comparisons could have resulted in increased type I errors. The data we present on hormonal differences are meant to raise hypotheses regarding possible underlying factors that explain the differences in breast cancer incidence between countries. While we adjusted for BMI and conducted a sensitivity analysis restricted to parous women, we were limited by lack of exhaustive information on lifestyle and environmental factors which may influence estrogen concentrations. Factors that possibly differ between countries could include diet, breastfeeding practices, stress, immune factors, sanitation, exposure to livestock, and others. It is possible that adjustment for BMI did not account for differences in fat distribution or other aspects of body fatness. Furthermore, the distribution of age differed among groups as did the distribution of cycle day since over two thirds of British women donated samples during the peri-ovulatory phase. Reduced power owing to fewer samples in the luteal phase could explain the lack of significant findings.

There were also differences among the collection protocols of the Mongolian, Chinese, and British studies. First, urine samples were stored using ascorbic acid in the Shanghai Breast Cancer Study, while samples were frozen without preservative in the Mongolian and British samples. Previous stability studies conducted by the laboratory used for this study have shown little difference in concentrations of samples preserved with and without ascorbic acid [21]. Second, samples collected in Mongolia and the UK were stored for a similar length of time while storage time differed by 12 years between China and Mongolia. While no studies have tested the effects of length of storage on EM for this duration, prior work has demonstrated that sex steroid hormone concentrations are reliable over long-term storage [22–24]. In particular, EM are relatively stable after being stored for 1 year and after a 2 to 3 year period [21]. Hormone values declined less than 1.2% with longer storage time [25], a level of degradation not sufficient to alter our ability to detect population differences.

## Conclusion

In conclusion, the metabolism of estrogens, mainly estradiol, results in products with reduced estrogenic activity [26]. Our data showing the possibility of greater estrogen metabolism in women living in Mongolia, with one of the lowest breast cancer rates globally, than in women living in China, and in Chinese compared with British women, is consistent with their relative breast cancer risks and suggestive of a potentially protective metabolic profile. 

## Supplementary Information

Supplementary Material 1.

Supplementary Material 2.

Supplementary Material 3. Supplemental Table 1. Adjusted least square means for selected metabolites (pg/mL per mg of creatinine).

## Data Availability

To request data, please contact Dr. Ganmaa Davaasambuu at gdavaasa@hsph.harvard.edu.

## Abbreviations

U.S: United States
U.K: United Kingdom
BMI: Body mass index
IARC: International Agency for Research on Cancer
EM: Estrogen metabolites
