# Supplementary material for: Estrogen metabolites in premenopausal women living in China, Mongolia, and the United Kingdom
Source: BMC Cancer. 2026 Jun 10;26:1000. doi: 10.1186/s12885-026-16169-x (PMC13491713; doi:10.1186/s12885-026-16169-x)
Supplement: Supplementary file 1 [file 12885_2026_16169_MOESM2_ESM.pdf]

British Breast Cancer Study (NCRN)

Non-Communicable Disease Epidemiology Unit
London School of Hygiene and Tropical Medicine
University of London
Keppel Street
London
WC1E 7HT

Section of Epidemiology
The Institute of Cancer Research
Brookes Lawley Building
15 Cotswold Road
Sutton, Surrey
SM2 5NG

CONFIDENTIAL

Thank you for agreeing to participate in this study. We are most grateful for your help. May we remind you again that if you decide to withdraw from this research at any time you are completely free to do so.

Confidentiality

We would like to stress again that your answers will be treated in the strictest confidence and will not be disclosed to any third party, including your doctors. The information you provide will be used only for statistical analysis. It will not be possible to identify individuals in any results.

How to answer

Just put a tick in the box under the appropriate answer, like this [√]

OR write a number in the box, like this [| | |]

OR write details on a line, like this ……………………………………………………

If you do not know the answer to a question, or cannot remember please put "**NK**" (meaning **N**ot **K**nown).

If a question does not apply to you please put "**NA**" (meaning **N**ot **A**pplicable).

If we have not given you enough room for some of your answers, please use a blank sheet.

Date of completion: ……./……./……...

GENERAL INFORMATION ABOUT YOURSELF

1 What is your full name? ……………………………………………………………………

2 What is your address? ……………………………………………………………………

……………………………………………………………………

……………………………………………………………………

……………………………………………………………………

3 What is your telephone number? ……………………………………………………………

4 What is the name of your GP? ……………………………………………………………

5 What is the address of your GP's surgery? ……………………………………………

……………………………………………

……………………………………………

……………………………………………

6 What is the telephone number of your GP's surgery? ……………………………………………

7 What is your date of birth? day ☐☐ month ☐☐ year ☐☐☐☐

8 What is your height? feet ☐ inches ☐☐ (or in cm) ☐☐☐

9 What is your current weight? stones ☐☐ lbs ☐☐ (or in Kg) ☐☐☐

10 How would you describe your ethnic origin *(tick one box)*

☐ White
☐ Jewish
☐ Indian
☐ Pakistani
☐ Bangladeshi
☐ Chinese
☐ Black-Caribbean
☐ Black-African
☐ Other

If other, please specify ………………………. …………………………

INFORMATION ABOUT YOUR HEALTH

11 Have you ever smoked cigarettes? ☐ yes ☐ no

If yes, do you still smoke? ☐ yes ☐ no

For how many years have you ever smoked? ☐☐ years

And, on average, how many cigarettes did/do you smoke per day? ☐☐

12 Have you ever been told by a doctor that you have diabetes? ☐ yes ☐ no

If yes, are you **currently taking** any medication for your diabetes? ☐ yes ☐ no

If yes, please give the name(s) of the medication(s) you are **currently taking**

……………………………………………………………………………………………………

……………………………………………………………………………………………………

13 How old were you when your periods began? ☐☐ years old

☐ never had periods

14 Are you still having periods? ☐ yes ☐ no

If yes, are they ☐ regular ☐ irregular

15 If you are no longer having periods, how old were you when they stopped completely? ☐☐ years old

And, did they stop as a result of surgery (e.g. hysterectomy or removal of ovaries) ? ☐ yes ☐ no

16 Have you ever used the contraceptive pill? ☐ yes ☐ no

If yes, for how long, in total? ☐☐ years ☐☐ months

And are you currently taking the contraceptive pill? ☐ yes ☐ no

17 Have you ever used hormone replacement therapy (HRT) ? ☐ yes ☐ no

If yes, for how long in total? ☐☐ years ☐☐ months

And, are you currently using hormone replacement therapy? ☐ yes ☐ no

18 Have you ever been diagnosed with any type of cancer? ☐ yes ☐ no

If yes, please give the type of cancer ……………………………………… …………….

How old were you when this cancer was first diagnosed? ☐☐ years old

19 Have you ever had a mammogram? ☐ yes ☐ no

If yes, when did you have your **first** mammogram?
(*please tell us your age or the year –whichever is easier)*

age ☐☐ year ☐☐☐☐

20 Was your **first** invitation for a mammogram sent as part of the national screening programme? ☐ yes ☐ no

If no, who advised you to have your first mammogram?

General practitioner (GP) ☐ Occupational/private medical ☐

Consultant in clinical genetics ☐ Other ……………………………………………..

Why were you referred for a mammogram?

……………………………………………………………………………………………………

21 When and where did you have your **last** mammogram? month ☐☐ year ☐☐☐☐

Name of screening unit/hospital ……………………………………………………………...

Town ……………………………………………………………………

22 In total, how many mammograms have you had? ☐☐ mammograms

And, approximately how often do you have a mammogram?

☐ every 3 years

☐ every 2 years

☐ every year

23 Was any abnormality ever detected? ☐ yes ☐ no

If yes, please give details ……………………………………………………………………..

……………………………………………………………………………………………………

FAMILY HISTORY

We would like to ask you some questions about your **blood relatives**. Please only include details about your natural parents, children who are genetically related to you (not adopted children or step-children). We ask for this information because we are trying to understand about cancer in families. *We will not use this information to try to contact any of your relatives.*

24 Have any of your parents or grand parents been diagnosed with cancer? ☐ yes ☐ no ☐ don't know

If yes, what was their relationship to you?

Mother ☐ Type of cancer ……………………………………………………

Father ☐ Type of cancer ……………………………………………………

Grandmother (maternal) ☐ Type of cancer ……………………………………………………

Grandfather (maternal) ☐ Type of cancer ……………………………………………………

Grandmother (paternal) ☐ Type of cancer ……………………………………………………

Grandfather (paternal) ☐ Type of cancer ……………………………………………………

25 How many children do you have/have you had? ☐☐
(please enter "0" if you have never had children)

Are you currently pregnant? ☐ yes ☐ no

Are you currently breastfeeding? ☐ yes ☐ no

Have any of your children ever had cancer? ☐ yes ☐ no

If yes, please specify the type of cancer? …………………………………………………………...

26 How many **full** sisters and brothers do you have/have you had? ☐☐
(please enter "0" if you have none)

Have any of your full sisters or brothers ever have cancer? ☐ yes ☐ no

If yes, please specify the type of cancer ……………………………......................................

27 Have any other of your close blood relatives been diagnosed with cancer?

	Yes	No	Don't know	Maternal (mother's side)	Paternal (father's side)	Type of cancer
Aunt	☐	☐	☐	☐	☐	…………………..
Uncle	☐	☐	☐	☐	☐	…………………..
Niece	☐	☐	☐	☐	☐	…………………..
Nephew	☐	☐	☐	☐	☐	……………........
First cousin	☐	☐	☐	☐	☐	…………………..
Half-brother	☐	☐	☐	☐	☐	…………………..
Half-sister	☐	☐	☐	☐	☐	…………………..

Thank you for taking the time to complete this questionnaire. If there were any questions you did not understand, or if you have any questions about the study, please do not hesitate to contact one of the study organisers whose names and contact details are given on the inside cover of the questionnaire.

If we have any queries about the answers you have given in this questionnaire it would be helpful if we could phone you to clarify them. If however, you do not wish us to contact you, please say so.

You **may*/may not*** contact me to clarify any of my answers which you are not clear about. (*please delete as appropriate)

Thank you once again. We are very grateful for your help.

Finally, would you now please sign and date this questionnaire.

Signed……………………………………………………

Date………………………………………………………
