# Supplementary material for: Estrogen metabolites in premenopausal women living in China, Mongolia, and the United Kingdom
Source: BMC Cancer. 2026 Jun 10;26:1000. doi: 10.1186/s12885-026-16169-x (PMC13491713; doi:10.1186/s12885-026-16169-x)
Supplement: Supplementary file 2 [file 12885_2026_16169_MOESM1_ESM.docx]

**Appendix 4:**

QUESTIONNAIRE AMONG THE ULAANBAATAR SCHOOL MOMS

___	___	/ ___	___	/ ___ ___

YEAR	MONTH	DAY

**Thank you for joining the School Moms Study! Please fill out this form and provide us with necessary information. If you have any questions someone from our research team will help you.**

1.	What is your date of birth?	19 ___	___	/ ___	___	/ ___	___

YEAR	MONTH	DAY

2.	What is the highest grade in school you have completed (mark one box)?

1 ☐	None

2 ☐	1 to 6 years

3 ☐	7 to 10 but did not finish high school

4 ☐	Graduated high school

5 ☐	Some college or University

4 ☐	Graduated college or university

3.	What is your current marital status (mark one box)?

1 ☐ Single (never married)

2 ☐ Married

3 ☐ Divorced or separated

4 ☐ Widowed

4.	What kind of work are you currently doing? _____________________________

5.	What kind of occupation do you have?

6. Where do you currently live (mark one box)?

a.	⁪ ☐ Ger (yurt)

⁪ ☐ Apartment (please write the number of the rooms)______________

⁪ ☐ House (connected to the central hearing system)

⁪ ☐	House non connected to the central heating system (sub-urban style)

b.	How many people do you live with? _________

NUMBER

c.	Of the people you live with, how many have full-time jobs? ____

NUMBER

d.	What kinds of jobs do they have?

Relation with you________________________________

Kind of job_____________________________________

A. Husband____________________________________

B. Father______________________________________

C. Mother______________________________________

e.	Your address:

Ulaanbaatar city	______________ district; ____________khoroo; ____________

f.	How long have you lived on this address? ______ or __________

YEARS	MONTHS

g.	Have you lived anywhere before your current address? If so, what was your

i. Ulaanbaatar city, District; horoo:______________________________________

ii. ……………………………….providence………………………..sum

h. Were you a nomad?

1 ☐ YES	2 ☐ NO

7.	Do your family own or drive a car?

1 ☐ YES	2 ☐ NO

Now we will ask you about your childhood.

8.	Where were you born? ________________/city, aimag(providence)/_________________/district, sum/

9.	What kind of work did your parents do?___________________________________

_________________________________________________________________

10.	Did your family spend summers on the huduu (to the countryside)?

1 ☐ YES	2 ☐ NO

11. Does your family go to the summer place in the summer?

1 ☐ YES	2 ☐ NO

12.	Was there a television in your home when you were a child?

1 ☐ YES	2 ☐ NO

The next set of questions has to do with your reproductive experience.

13.	How old were you when you had your first period?	AGE

14.	During the past 12 months, did you ever go for more than six weeks without having a menstrual period? (Please don't count the times your periods stopped because you were pregnant, breast-feeding or using oral contraceptives.)

1 ☐ YES	2 ☐ NO

15.	Have you ever used birth control pills, injections or Norplant?

1 ☐ YES	2 ☐ NO **GO TO QUESTION 16**

a.	Are you currently using birth control pills, injections or Norplant?

1 ☐ YES	2 ☐ NO

16.	Have you ever tried to become pregnant for 12 months or more without success?

1 ☐ YES	2 ☐ NO

17.	Pregnancy:

a.	How many times, in total, have you been pregnant?	_____________

PREGNANCIES

b.	How many of these pregnancies resulted in:

Live births _________

NUMBER

How old were you when you had your first live birth? ______

YEARS

What was the date of your last live birth?

___	___	/ ___	___	/ ___ ___

YEAR	MONTH	DAY

What was the weight of your heaviest baby? _______

GRAMS

Miscarriages (pregnancy ended before 24 weeks) ________

NUMBER

Abortions __________

NUMBER

Still births (pregnancy ended at or after 24 weeks) __________

NUMBER

Ectopic pregnancies (in the fallopian tube) ___________

NUMBER

18.	Did you breastfeed any of your children?

1 ☐ YES	2 ☐ NO **GO TO QUESTION 19**

a.	How many months did you breastfeed each of your children?

_____ child 1	_____ child 3	_____ child 5	_____ child 7

MONTHS	MONTHS	MONTHS	MONTHS

_____ child 2	_____ child 4	_____ child 6	_____ child 8

MONTHS	MONTHS	MONTHS	MONTHS

b.	How recently did you stop breastfeeding your youngest child?

_______	__________

YEARS	MONTHS

The next set of questions has to do with your medical history.

19.	What was your weight when you were 18 years of age (or if you were pregnant, your usual weight before the pregnancy)? _______

KG

20.	Have you ever been diagnosed as having any type of cancer?

1 ☐ YES	2 ☐ NO **GO TO QUESTION 21**

| Type of Cancer  (Where cancer started) | Year first diagnosed | Name and Address of Doctor/Hospital  Where Cancer was Diagnosed |
| --- | --- | --- |
|  |  |  |
|  |  |  |

21.	Have any of your mother, aunts or sisters been diagnosed with breast cancer?

1 ☐ YES	2 ☐ NO

22.	Were you (yourself) born (mark one box):

⁪ ☐ very small?

⁪ ☐ small?

⁪ ☐ average?

⁪ ☐ big?

⁪ ☐ very big?

23. Do you know what you weighed at birth?

1 ☐ YES	2 ☐ NO **GO TO QUESTION 24**

___________

GRAMS

The next set of questions has to do with your lifestyle.

24.	Have you ever smoked cigarettes regularly for six months or longer (one or more

cigarette a day)?

1 ☐ YES	2 ☐ NO **GO TO QUESTION 25**

a.	At what age did you start smoking cigarettes regularly? _

AGE

b.	Do you currently smoke cigarettes?

1 ☐ YES	2 ☐ NO

c. Stopped smoking

☐	At what age did you stop? ____

AGE

25.	Do you consume alcoholic beverages?

1 ☐ YES	2 ☐ NO **GO TO QUESTION 26**

a.	Do you drink: (check one box)

☐ At least 1 drink per day?

☐ 4-6 drinks per week?

☐ 1-3 drinks per week?

☐ Less than 1 drink per week?

**These next questions ask about the time you spend doing different types of physical activity in a typical week. Please answer these questions even if you do not consider yourself to be a physically active person. Think first about the time you spend doing work. Think of work as the things that you have to do such as paid or unpaid work, study/training, household chores, harvesting food/crops, fishing or hunting for food, seeking employment. In answering the following questions 'vigorous-intensity activities' are activities that require hard physical effort and cause large increases in breathing or heart rate, 'moderate-intensity activities' are activities that require moderate physical effort and cause small increases in breathing or heart rate.**

26.	Does your work involve vigorous-intensity activity that causes large increases in breathing or heart rate like [carrying or lifting heavy loads, digging or construction work] for at least 10 minutes continuously?

1 ☐ YES	2 ☐ NO **go to question 27**

27.	In a typical week, on how many days do you do vigorous-intensity activities as part of your work?	________________

NUMBER OF DAYS

28.	How much time do you spend doing vigorous-intensity activities at work on a typical day?	________	_________

HOURS and	MINUTES

29.	Does your work involve moderate-intensity activity that causes small increases in breathing or heart rate such as brisk walking [or carrying light loads] for at least 10 minutes continuously?

1 ☐ YES	2 ☐ NO **go to question 30**

30.	In a typical week, on how many days do you do moderate-intensity activities as part of your work?	________________

NUMBER OF DAYS

31.	How much time do you spend doing moderate-intensity activities at work on a typical day?	________	_________

HOURS and	MINUTES

**The next questions exclude the physical activities at work that you have already mentioned. These questions ask about the usual way you travel to and from places. For example to work, for shopping, to market, to place of worship.**

32.	Do you walk or use a bicycle (pedal cycle) for at least 10 minutes continuously to get to and from places?

1 ☐ YES	2 ☐ NO **go to question 33**

33.	In a typical week, on how many days do you walk or bicycle for at least 10 minutes continuously to get to and from places?	________________

NUMBER OF DAYS

34.	How much time do you spend walking or bicycling for travel on a typical day?

________	_________

HOURS and	MINUTES

**The next questions exclude the work and transport activities that you have already mentioned. These questions ask about sports, fitness and recreational (leisure) activities.**

35.	Do you do any vigorous-intensity sports, fitness or recreational (leisure) activities that cause large increases in breathing or heart rate like [running or football,] for at least 10 minutes continuously?

1 ☐ YES	2 ☐ NO **go to question 36**

36.	In a typical week, on how many days do you do vigorous-intensity sports, fitness or recreational (leisure) activities?	________________

NUMBER OF DAYS

37.	How much time do you spend doing vigorous-intensity sports, fitness or recreational activities on a typical day? ________	_________

HOURS and	MINUTES

38.	Do you do any moderate-intensity sports, fitness or recreational (leisure) activities that causes a small increase in breathing or heart rate such as brisk walking,(cycling, swimming, volleyball)for at least 10 minutes continuously?

1 ☐ YES	2 ☐ NO **go to question 39**

39.	In a typical week, on how many days do you do moderate-intensity sports, fitness or recreational (leisure) activities?	________________

NUMBER OF DAYS

40.	How much time do you spend doing moderate-intensity sports, fitness or recreational (leisure) activities on a typical day?	________	_________

HOURS and	MINUTES

**The following question is about sitting or reclining at work, at home, getting to and from places, or with friends including time spent [sitting at a desk, friends, traveling in car, bus, train, reading, playing cards or watching television], but do not include time spent sleeping.**

41.	How much time do you usually spend sitting or reclining on a typical day?

________	_________

HOURS and	MINUTES

42.	The next set of questions has to do with what you usually eat.

When you think about the regular past week(tsagaan sar and other celebrations not included), how often did you eat the following foods?

| Food Group | Food Item | Number of times  consumed  in past week  (write number) |
| --- | --- | --- |
| In the past week, how many times did you eat  meat and meat products? | Animal meat |  |
|  | Animal **organ** meat, liver or by-products |  |
|  | Chicken and pork |  |
|  | Fish, canned fish |  |
|  | Eggs |  |
|  | Other, specify: |  |
| In the past week, how many times did you eat  milk and dairy products? | Milk |  |
|  | Mongolian yogurt |  |
|  | **Imported** and packaged yogurt |  |
|  | Sour milk |  |
|  | Dried curd |  |
|  | Mongolian cheese |  |
|  | Other cheese |  |
|  | Other, specify: |  |
| In the past week, how many times did you eat  milk, cream and butter? | Milk cream |  |
|  | Sour cream |  |
|  | Clotted cream |  |
|  | Milk butter |  |
|  | Butter |  |
| In the past week, how many times did you eat  vegetables? | Green onion |  |
|  | Chinese cabbage |  |
|  | Pepper |  |
|  | Tomato |  |
|  | Turnip |  |
|  | Carrot |  |
|  | Cucumber |  |
|  | Potato |  |
|  | Other, specify: |  |
| In the past week, how many times did you eat flour products? |  |  |
| In the past week, how many times did you eat rice? |  |  |
| In the past week, how many times did you eat fruit? |  |  |
| In the past week, how many times did you drink fruit juice? |  |  |

43.	How many cups per day, do you drink tea?	________

NUMBER

If you drink tea, what do you usually drink? (Mark all that apply.)

☐Ordinary milk tea

☐Milk tea with rice and fat

☐Black tea

☐Green tea

☐ Other, please specify: _____________________

44.	Do you take any vitamins or dietary supplements?

1 ☐ YES	2 ☐ NO

What do you take (mark all that apply)?

☐ multiple vitamin

☐ vitamin D

☐ vitamin C

☐ Other, specify: ________________

**Thank you for taking the time**

**to complete this questionnaire.**
