# Supplementary material for: Estrogen metabolites in premenopausal women living in China, Mongolia, and the United Kingdom
Source: BMC Cancer. 2026 Jun 10;26:1000. doi: 10.1186/s12885-026-16169-x (PMC13491713; doi:10.1186/s12885-026-16169-x)
Supplement: Supplementary file 3 [file 12885_2026_16169_MOESM3_ESM.docx]

**Supplemental Table 1. Adjusted least square means for selected metabolites (pg/mL per mg of creatinine)**

|  | **Adjusted for age and cycle day** | | | | |  | **Adjusted for age, cycle day, BMI, and parity** | | | | | |
| --- | --- | --- | --- | --- | --- | --- | --- | --- | --- | --- | --- | --- |
|  | British | | Chinese | | Mongolian | | British | | Chinese | | Mongolian | |
|  | Mean | (SD) | Mean | (SD) | Mean | (SD) | Mean | (SD) | Mean | (SD) | Mean | (SD) |
| **Total** |  |  |  |  |  |  |  |  |  |  |  |  |
| Follicular | 8.001 | (0.124) | 7.437 | (0.117) | 7.561 | (0.102) | 8.006 | (0.128) | 7.392 | (0.125) | 7.545 | (0.104) |
| Peri-ovulatory | 8.119 | (0.072) | 7.850 | (0.174) | 7.690 | (0.109) | 8.102 | (0.077) | 7.823 | (0.179) | 7.697 | (0.112) |
| Luteal | 7.638 | (0.212) | 7.258 | (0.132) | 7.325 | (0.115) | 7.840 | (0.253) | 7.261 | (0.137) | 7.356 | (0.119) |
| **Estrone** |  |  |  |  |  |  |  |  |  |  |  |  |
| Follicular | 6.629 | (0.152) | 6.087 | (0.144) | 5.843 | (0.126) | 6.679 | (0.158) | 6.045 | (0.154) | 5.834 | (0.128) |
| Peri-ovulatory | 6.769 | (0.088) | 6.514 | (0.215) | 5.888 | (0.134) | 6.722 | (0.095) | 6.490 | (0.220) | 5.904 | (0.138) |
| Luteal | 5.576 | (0.261) | 5.690 | (0.163) | 5.445 | (0.141) | 5.672 | (0.311) | 5.689 | (0.168) | 5.480 | (0.146) |
| **Estradiol** |  |  |  |  |  |  |  |  |  |  |  |  |
| Follicular | 6.137 | (0.148) | 5.344 | (0.140) | 5.297 | (0.122) | 6.187 | (0.153) | 5.323 | (0.149) | 5.292 | (0.124) |
| Peri-ovulatory | 6.197 | (0.086) | 5.814 | (0.208) | 5.386 | (0.130) | 6.157 | (0.092) | 5.804 | (0.214) | 5.398 | (0.134) |
| Luteal | 5.425 | (0.254) | 5.043 | (0.158) | 4.873 | (0.137) | 5.627 | (0.302) | 5.044 | (0.163) | 4.893 | (0.142) |
| **2-Pathway (total)** |  |  |  |  |  |  |  |  |  |  |  |  |
| Follicular | 6.317 | (0.145) | 5.830 | (0.137) | 5.843 | (0.120) | 6.320 | (0.150) | 5.772 | (0.146) | 5.823 | (0.122) |
| Peri-ovulatory | 6.601 | (0.084) | 5.967 | (0.204) | 5.923 | (0.128) | 6.620 | (0.091) | 5.926 | (0.209) | 5.925 | (0.131) |
| Luteal | 6.342 | (0.249) | 5.477 | (0.155) | 5.609 | (0.135) | 6.499 | (0.296) | 5.476 | (0.160) | 5.642 | (0.139) |
| **3-ME1** |  |  |  |  |  |  |  |  |  |  |  |  |
| Follicular | 2.665 | (0.172) | 2.203 | (0.163) | 2.431 | (0.143) | 2.632 | (0.179) | 2.167 | (0.175) | 2.448 | (0.146) |
| Peri-ovulatory | 2.816 | (0.100) | 2.235 | (0.243) | 2.555 | (0.152) | 2.813 | (0.109) | 2.201 | (0.251) | 2.580 | (0.157) |
| Luteal | 2.209 | (0.296) | 2.375 | (0.184) | 2.421 | (0.160) | 1.998 | (0.354) | 2.343 | (0.191) | 2.442 | (0.167) |
| **2-methoxyestrone** |  |  |  |  |  |  |  |  |  |  |  |  |
| Follicular | 5.237 | (0.161) | 4.773 | (0.153) | 4.597 | (0.133) | 5.259 | (0.167) | 4.736 | (0.163) | 4.586 | (0.136) |
| Peri-ovulatory | 5.427 | (0.093) | 5.049 | (0.227) | 4.724 | (0.142) | 5.428 | (0.101) | 5.018 | (0.233) | 4.723 | (0.146) |
| Luteal | 5.039 | (0.277) | 4.341 | (0.172) | 4.429 | (0.150) | 5.242 | (0.330) | 4.336 | (0.178) | 4.448 | (0.155) |
| **2-methoxyestradiol** |  |  |  |  |  |  |  |  |  |  |  |  |
| Follicular | 2.675 | (0.259) | 2.056 | (0.245) | 2.040 | (0.214) | 2.646 | (0.268) | 2.037 | (0.261) | 2.040 | (0.218) |
| Peri-ovulatory | 2.474 | (0.150) | 1.802 | (0.365) | 2.269 | (0.228) | 2.459 | (0.162) | 1.775 | (0.374) | 2.262 | (0.235) |
| Luteal | 2.801 | (0.444) | 1.893 | (0.276) | 1.789 | (0.240) | 3.013 | (0.529) | 1.877 | (0.286) | 1.786 | (0.249) |
| **2-hydroxyestrone** |  |  |  |  |  |  |  |  |  |  |  |  |
| Follicular | 5.367 | (0.167) | 4.900 | (0.158) | 5.038 | (0.138) | 5.355 | (0.173) | 4.839 | (0.168) | 5.006 | (0.140) |
| Peri-ovulatory | 5.816 | (0.097) | 5.076 | (0.236) | 5.086 | (0.147) | 5.849 | (0.104) | 5.036 | (0.241) | 5.079 | (0.151) |
| Luteal | 5.559 | (0.287) | 4.627 | (0.179) | 4.632 | (0.155) | 5.708 | (0.341) | 4.638 | (0.184) | 4.666 | (0.160) |
| **2-hydroxyestradiol** |  |  |  |  |  |  |  |  |  |  |  |  |
| Follicular | 4.336 | (0.177) | 3.462 | (0.168) | 3.923 | (0.146) | 4.385 | (0.184) | 3.415 | (0.179) | 3.885 | (0.149) |
| Peri-ovulatory | 4.483 | (0.103) | 3.470 | (0.249) | 3.996 | (0.156) | 4.530 | (0.111) | 3.445 | (0.256) | 3.984 | (0.161) |
| Luteal | 4.395 | (0.304) | 2.925 | (0.189) | 3.613 | (0.164) | 4.543 | (0.363) | 2.948 | (0.196) | 3.641 | (0.170) |
| **16-pathway (total)** |  |  |  |  |  |  |  |  |  |  |  |  |
| Follicular | 6.845 | (0.147) | 6.342 | (0.139) | 6.806 | (0.122) | 6.823 | (0.152) | 6.313 | (0.148) | 6.782 | (0.124) |
| Peri-ovulatory | 6.860 | (0.085) | 6.765 | (0.207) | 6.913 | (0.130) | 6.863 | (0.092) | 6.749 | (0.212) | 6.905 | (0.133) |
| Luteal | 6.645 | (0.252) | 6.256 | (0.157) | 6.521 | (0.137) | 6.853 | (0.300) | 6.270 | (0.162) | 6.538 | (0.141) |
| **16a-hydroxyestradiol** |  | |  |  |  |  |  |  |  |  |  |  |
| Follicular | 5.015 | (0.176) | 4.731 | (0.167) | 4.785 | (0.146) | 4.926 | (0.181) | 4.587 | (0.177) | 4.796 | (0.148) |
| Peri-ovulatory | 5.045 | (0.102) | 5.155 | (0.248) | 4.730 | (0.155) | 4.983 | (0.110) | 5.039 | (0.254) | 4.791 | (0.159) |
| Luteal | 4.390 | (0.302) | 4.358 | (0.188) | 4.014 | (0.163) | 4.521 | (0.358) | 4.296 | (0.194) | 4.106 | (0.168) |
| **16keto-estradiol** |  |  |  |  |  |  |  |  |  |  |  |  |
| Follicular | 4.294 | (0.156) | 4.160 | (0.147) | 4.646 | (0.129) | 4.254 | (0.160) | 4.061 | (0.156) | 4.639 | (0.130) |
| Peri-ovulatory | 4.320 | (0.090) | 4.509 | (0.220) | 4.642 | (0.137) | 4.345 | (0.097) | 4.428 | (0.224) | 4.666 | (0.140) |
| Luteal | 4.409 | (0.267) | 4.014 | (0.166) | 4.335 | (0.145) | 4.420 | (0.316) | 3.983 | (0.171) | 4.392 | (0.149) |
| **16EpiEstriol** |  |  |  |  |  |  |  |  |  |  |  |  |
| Follicular | 5.007 | (0.151) | 4.328 | (0.144) | 4.659 | (0.125) | 4.995 | (0.157) | 4.310 | (0.153) | 4.645 | (0.127) |
| Peri-ovulatory | 4.998 | (0.088) | 4.687 | (0.214) | 4.658 | (0.134) | 4.984 | (0.095) | 4.679 | (0.219) | 4.656 | (0.137) |
| Luteal | 4.830 | (0.260) | 4.123 | (0.162) | 4.310 | (0.141) | 5.227 | (0.309) | 4.130 | (0.167) | 4.321 | (0.145) |
| **17EpiEstriol** |  |  |  |  |  |  |  |  |  |  |  |  |
| Follicular | 3.265 | (0.208) | 2.984 | (0.197) | 2.681 | (0.172) | 3.208 | (0.214) | 2.835 | (0.209) | 2.703 | (0.174) |
| Peri-ovulatory | 3.269 | (0.120) | 3.704 | (0.293) | 2.726 | (0.183) | 3.234 | (0.130) | 3.574 | (0.299) | 2.786 | (0.188) |
| Luteal | 3.719 | (0.357) | 3.135 | (0.222) | 2.593 | (0.193) | 3.847 | (0.423) | 3.058 | (0.229) | 2.682 | (0.199) |
| **Estriol** |  |  |  |  |  |  |  |  |  |  |  |  |
| Follicular | 6.171 | (0.157) | 5.564 | (0.149) | 6.224 | (0.130) | 6.165 | (0.162) | 5.585 | (0.158) | 6.191 | (0.132) |
| Peri-ovulatory | 6.170 | (0.091) | 6.025 | (0.222) | 6.421 | (0.139) | 6.194 | (0.098) | 6.053 | (0.227) | 6.389 | (0.142) |
| Luteal | 5.954 | (0.270) | 5.606 | (0.168) | 6.085 | (0.146) | 6.174 | (0.320) | 5.646 | (0.173) | 6.069 | (0.151) |
| **4-pathway (total)** |  |  |  |  |  |  |  |  |  |  |  |  |
| Follicular | 3.945 | (0.158) | 3.713 | (0.150) | 3.818 | (0.131) | 3.904 | (0.164) | 3.608 | (0.159) | 3.824 | (0.133) |
| Peri-ovulatory | 4.407 | (0.092) | 3.776 | (0.224) | 3.963 | (0.140) | 4.394 | (0.099) | 3.690 | (0.228) | 4.002 | (0.143) |
| Luteal | 4.164 | (0.272) | 3.430 | (0.169) | 3.649 | (0.147) | 4.502 | (0.323) | 3.386 | (0.174) | 3.715 | (0.152) |
| **4-hydroxyestrone** |  |  |  |  |  |  |  |  |  |  |  |  |
| Follicular | 3.591 | (0.181) | 3.481 | (0.171) | 3.692 | (0.150) | 3.553 | (0.187) | 3.379 | (0.182) | 3.705 | (0.152) |
| Peri-ovulatory | 4.279 | (0.105) | 3.639 | (0.255) | 3.808 | (0.159) | 4.262 | (0.113) | 3.551 | (0.261) | 3.849 | (0.163) |
| Luteal | 3.904 | (0.310) | 3.070 | (0.193) | 3.417 | (0.168) | 4.328 | (0.368) | 3.019 | (0.199) | 3.478 | (0.173) |
| **4-methoxyestradiol** |  |  |  |  |  |  |  |  |  |  |  |  |
| Follicular | 0.948 | (0.240) | 0.120 | (0.227) | 0.152 | (0.199) | 0.943 | (0.247) | 0.102 | (0.241) | 0.115 | (0.201) |
| Peri-ovulatory | 0.487 | (0.139) | -0.054 | (0.338) | 0.625 | (0.212) | 0.448 | (0.149) | -0.046 | (0.345) | 0.616 | (0.216) |
| Luteal | 1.079 | (0.412) | 0.434 | (0.256) | 0.231 | (0.223) | 1.173 | (0.488) | 0.474 | (0.264) | 0.255 | (0.229) |
| **4-methoxyestrone** |  |  |  |  |  |  |  |  |  |  |  |  |
| Follicular | 1.472 | (0.188) | 0.855 | (0.178) | 1.032 | (0.156) | 1.382 | (0.193) | 0.818 | (0.188) | 1.022 | (0.157) |
| Peri-ovulatory | 1.082 | (0.109) | 0.664 | (0.265) | 1.191 | (0.166) | 1.170 | (0.117) | 0.633 | (0.270) | 1.191 | (0.169) |
| Luteal | 1.244 | (0.323) | 0.667 | (0.201) | 1.036 | (0.175) | 1.385 | (0.381) | 0.657 | (0.206) | 1.049 | (0.179) |
| **Pathways and Ratios** |  | |  |  |  |  |  |  |  |  |  |  |
| **2:16 Pathway** |  |  |  |  |  |  |  |  |  |  |  |  |
| Follicular | -0.529 | (0.149) | -0.513 | (0.141) | -0.964 | (0.123) | -0.503 | (0.154) | -0.541 | (0.150) | -0.959 | (0.125) |
| Peri-ovulatory | -0.259 | (0.086) | -0.797 | (0.210) | -0.990 | (0.131) | -0.243 | (0.093) | -0.824 | (0.216) | -0.980 | (0.135) |
| Luteal | -0.303 | (0.256) | -0.779 | (0.159) | -0.912 | (0.139) | -0.354 | (0.305) | -0.794 | (0.165) | -0.896 | (0.143) |
| **Non-parent:parent** |  |  |  |  |  |  |  |  |  |  |  |  |
| Follicular | 0.265 | (0.100) | 0.415 | (0.095) | 0.877 | (0.083) | 0.198 | (0.103) | 0.408 | (0.100) | 0.865 | (0.083) |
| Peri-ovulatory | 0.303 | (0.058) | 0.237 | (0.142) | 0.954 | (0.089) | 0.346 | (0.062) | 0.231 | (0.143) | 0.941 | (0.090) |
| Luteal | 1.005 | (0.172) | 0.667 | (0.107) | 1.104 | (0.093) | 1.034 | (0.203) | 0.674 | (0.109) | 1.101 | (0.095) |
| **2-pathway:Parent** |  |  |  |  |  |  |  |  |  |  |  |  |
| Follicular | -0.828 | (0.112) | -0.667 | (0.106) | -0.476 | (0.093) | -0.877 | (0.115) | -0.688 | (0.112) | -0.488 | (0.094) |
| Peri-ovulatory | -0.636 | (0.065) | -1.015 | (0.158) | -0.458 | (0.099) | -0.574 | (0.070) | -1.036 | (0.161) | -0.470 | (0.101) |
| Luteal | 0.057 | (0.192) | -0.656 | (0.120) | -0.292 | (0.104) | 0.044 | (0.228) | -0.656 | (0.123) | -0.289 | (0.107) |
| **4-pathway:Parent** |  |  |  |  |  |  |  |  |  |  |  |  |
| Follicular | -3.200 | (0.154) | -2.784 | (0.146) | -2.500 | (0.128) | -3.292 | (0.159) | -2.853 | (0.155) | -2.488 | (0.129) |
| Peri-ovulatory | -2.830 | (0.090) | -3.206 | (0.218) | -2.418 | (0.136) | -2.800 | (0.096) | -3.272 | (0.222) | -2.392 | (0.139) |
| Luteal | -2.121 | (0.265) | -2.704 | (0.165) | -2.252 | (0.144) | -1.952 | (0.314) | -2.746 | (0.170) | -2.216 | (0.148) |
| **16-pathway:Parent** |  |  |  |  |  |  |  |  |  |  |  |  |
| Follicular | -0.300 | (0.126) | -0.155 | (0.119) | 0.488 | (0.104) | -0.373 | (0.129) | -0.148 | (0.125) | 0.471 | (0.105) |
| Peri-ovulatory | -0.377 | (0.073) | -0.218 | (0.177) | 0.532 | (0.111) | -0.331 | (0.078) | -0.212 | (0.180) | 0.510 | (0.113) |
| Luteal | 0.360 | (0.216) | 0.123 | (0.134) | 0.620 | (0.117) | 0.398 | (0.254) | 0.138 | (0.137) | 0.607 | (0.119) |
